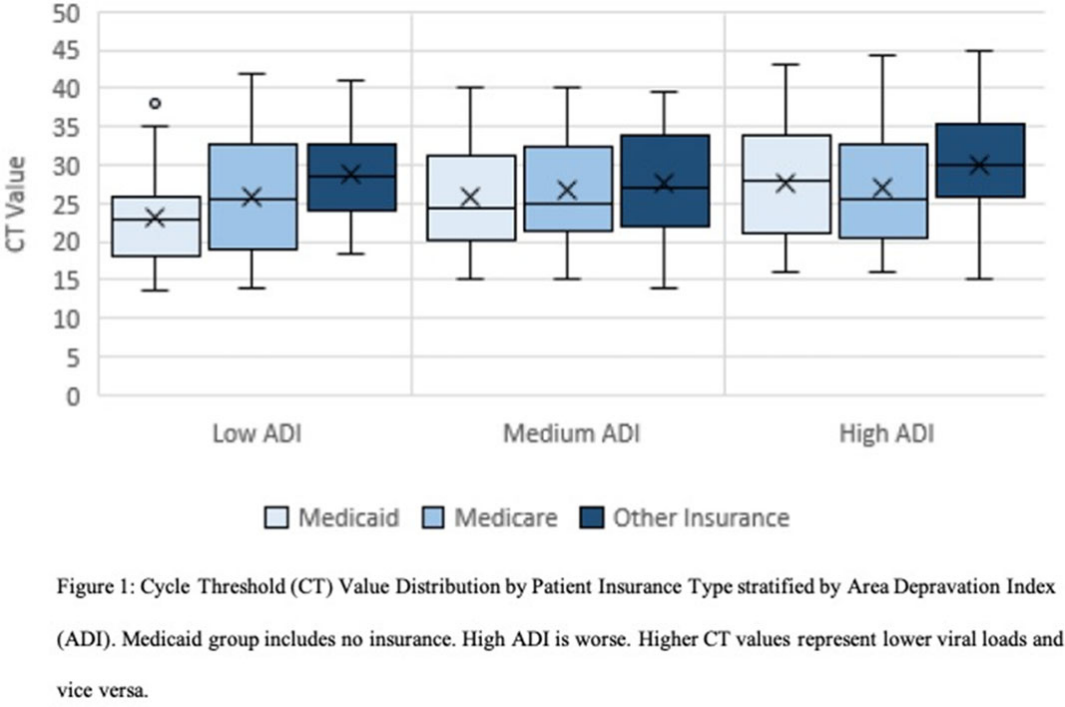# Sociodemographic Factors, Cycle Threshold Values, and Clinical Outcomes of COVID-19

**DOI:** 10.1017/ash.2021.33

**Published:** 2021-07-29

**Authors:** Frida Rivera, Cameron Gmehlin, Liliana Pezzin, Ann B. Nattinger, Ryan Hanson, Adriana Perez, Siddhartha Singh, Blake Buchan, Nathan Ledeboer, L. Silvia Munoz-Price

## Abstract

**Background:** The gold standard for diagnosis of COVID-19 has been SARS-CoV-2 detection by reverse-transcriptase-quantitative polymerase chain reaction (RT-qPCR), which provides a semiquantitative indicator of viral load (cycle threshold, Ct). Our research group previously described how African American race and poverty were associated with an increased likelihood of hospitalization due to COVID-19. We sought to characterize the relationship between Ct values and clinical outcomes while controlling for sociodemographic factors. **Methods:** We conducted a cross-sectional study of SARS-CoV-2–positive patients admitted to Froedtert Health between March 16 and June 1, 2020. Ct values were obtained by direct interrogation of either cobas SARS-CoV-2 or Cepheid Xpert Xpress platforms. Patient demographics, comorbidities, symptoms at admission, health insurance, and hospital course were collected using electronic medical records. A proxy for socioeconomic disadvantage, area-deprivation index (ADI), was assigned using ZIP codes. Multivariate models were performed to assess associations between Ct values and clinical outcomes while controlling for ADI, race, and type of insurance. **Results:** Overall, 302 patients were included. The mean age was 60.89 years (SD, 18.2); 161 (53%) were men, 177 (58%) were African Americans; and 156 (51%) had Medicaid or were uninsured. Of the 302 inpatients, 158 (52%) required admission to the ICU, 199 (65.9%) were discharged to home, 49 (16.2%) were discharged to a nursing home, and 54 (17.9%) died. Lower Ct values (higher viral load) were associated with Medicaid or lack of insurance (coefficient, −2.88, 95% confidence interval [CI], −4.96 to −0.79, *P* = .007) and age >60 years old (coefficient, −2.98, 95% CI −4.87 to −1.08, *P* = .002). Contrary to what was expected, higher CT values (lower viral load) were associated with higher ADI scores (coefficient, 2.62, 95% CI, 0.52–4.85; *P* = .017). However, when patients were stratified into low, medium, and high ADI, those with Medicaid or no insurance had the lowest mean Ct values (23.3, 25.9, and 27.6, respectively) compared to Medicare or other insurance (Figure 1). Body mass index (odds ratio [OR], 1.04; 95% CI, 1.02–1.07; *P* = .001) and male sex (OR, 2.15; 95% CI, 1.28–3.60; *P* = .004) were independently associated with ICU admission. Every increase of a CT point (OR, 0.90; 95% CI, 0.85–0.95; p <0.001) and age >60 years old (OR 2.62, 95% CI; 1.14-6.04; p=0.023) was associated with death. **Conclusions:** In this cross-sectional study of adults tested for COVID-19 in a large midwestern academic health system, lower Ct values were independently associated with poverty and age >60 years old.

**Funding:** No

**Disclosures:** None

**Figure 1.**